# The Impact of Wheat Growth Stages on Soil Microbial Communities in a Rain-Fed Agroecosystem

**DOI:** 10.3390/microorganisms13040838

**Published:** 2025-04-07

**Authors:** Yosef Steinberger, May Levi, Itaii Applebaum, Chen Sherman, Tirza Doniger, Adrian Unc

**Affiliations:** 1 The Mina & Everard Goodman Faculty of Life Sciences, Bar-Ilan University, Ramat Gan 5290002, Israel; may.levi@gmail.com (M.L.); itaiiapplebaum@gmail.com (I.A.); chen.sherman1@gmail.com (C.S.); tirza.doniger@biu.ac.il (T.D.); 2 School of Science and the Environment, Memorial University of Newfoundland, Corner Brook, NL A2H 5G4, Canada; aunc@mun.ca

**Keywords:** wheat rhizosphere, microbial activity, biomass, rain-fed management, wheat phenology

## Abstract

Wheat is the largest terrestrial agricultural crop globally. This study was conducted to determine the soil microbial biomass, soil CO_2_ evolution, and physiological profile in the rhizosphere of the winter wheat rain-fed *Triticum aestivum* along the development stages in a rain-fed semi-arid agro-ecosystem. The data show that a significant, over 100-fold increase in the utilization of four substrate groups (benzoic acid, amino acid, carbohydrates, and carboxylic acid) occurred in the wheat soil rhizosphere along the wheat growth phenology. After the stubble field stage, there was a notable decrease in the utilization of all four substrates. The occurrence of each substrate in the soil aligns with the below-ground rhythm of wheat plant biomass growth. The abundance of fine roots, categorizing wheat plant roots, in the soil at maturity and the stubble field stage may explain the heightened activity and diversity of copiotroph bacteria. This association suggests a potential link between the richness of fine roots and the increased activity and diversity of copiotroph bacteria in the soil. The findings clarify the impact of constraining abiotic factors, coupled with the phenological influences of wheat plants, and their combined effects on substrate utilization by microbial communities in a rain-fed *Triticum aestivum* wheat field.

## 1. Introduction

The soil is teeming with vital microbial populations, including bacteria, fungi, and nematodes, which form the foundation of the ecosystem’s food chain [1,2]. With an estimated range of 10^5^–10^9^ cells per gram of soil, this microflora orchestrates essential processes for soil fertility and provides crucial support to agricultural systems through various mechanisms [3,4]. As plants transition through different growth stages, they release organic matter through their roots, triggering shifts in the abiotic and biotic components of the rhizosphere system [5]. Root biomass and its exudates encompass an array of compounds that can regulate the soil microbial community, consequently influencing soil biota composition, trophic group organization, functionality, and even soil physico-chemical properties [6]. Alterations in abiotic soil properties, such as pH, moisture, and organic matter, influenced by the spatial heterogeneity of soil properties are correlated with soil respiration in response to various soil management practices [7,8]. Research by Griffiths et al. (1998) [9] and Landesman et al. (2011) [10] has consistently demonstrated that root biomass and the structure of the microflora community changes and have significant impacts on soil respiration where dense roots may promote autotrophic respiration. The ratio of root mass to shoot mass typically exceeds 0.4 and can soar to 3.7 in Mediterranean ecosystems [11]. This ratio not only enhances soil organic matter but also provides stability across a broad spectrum of environmental conditions. An amplified root/shoot ratio underscores the critical role of root development in above-ground biomass performance. The primary requirements along the stages of wheat growth are nitrogen, phosphate, potassium, sulfur, and manganese which contribute to early and rapid growth followed by enhancing root mass, yield quality, and photosynthetic proteins, respectively. The rhizosphere is profoundly influenced by the impact of roots on the soil’s physical structure, thereby shaping the interplay of physical, biological, and chemical processes. The timing of plant life cycles can impact the diversity and composition of soil microbial communities through several mechanisms, including changes in the quantity and composition of root exudates and hormones [12]. Consequently, the rhizosphere emerges as a pivotal site for soil microbial activity, nutrient cycling, and the decomposition of organic matter [13]. Prior investigations delved into how plant phenology affects the rhizosphere microbial community, often focusing on specific enzymes, root exudates, or the taxonomic microbial community composition.

Plant roots consistently release carbon dioxide (CO_2_) into the soil through carbohydrate respiration. Higher basal respiration rates suggest increased microbial metabolic activity, often reflecting greater decomposition of organic matter and nutrient cycling. Conversely, lower respiration levels may indicate reduced microbial activity due to environmental constraints such as limited moisture or nutrient availability [14]. Even minimal amounts of readily available substrates from root exudates effectively stimulate microbial biomass activity, leading to a significant increase in CO_2_ production. Furthermore, drought conditions definitively alter the composition of root exudates, resulting in notable changes in the activity of root-associated microorganisms.

This study investigates how the different growth stages of wheat plants, from germination to harvest, affect soil microbial biomass and substrate utilization in rain-fed agricultural soils. The study presents results obtained from soil sampling in a wheat field at various stages of plant growth, using the SIR (Substrate Induce Respiration) laboratory measurements method [15]. The objectives of the study are to (1) correlate the increase in wheat root and shoot biomass below ground during different stages of wheat phenological development with the functional state of microbial community in the soil rhizosphere using substrate-induced respiration (SIR), and (2) to assess the impact of growth stages on microbial function and its functional diversity.

It is thus hypothesized that the function and diversity of the rhizosphere microbial communities will respond to the various stages of wheat growth. The results of this study will provide a better understanding of the soil respiration variability along the different stages of wheat phenological development in a rain-fed agroecosystem.

## 2. Material and Methods

### 2.1. Study Site

The research site is situated in the agricultural fields of Kibbutz Be’erot Yitzhak, Israel, at coordinates 32°02′05.1″ N and 34°54′48.2″ E, with an elevation of 52 m above sea level. The region experiences an average annual rainfall of 489 mm and a Mediterranean climate. The mean minimum temperature in this area is 2.8 °C, while the mean maximum temperature is 36.5 °C. The wheat field and the control area have similar textures: 77% sand, 17% silt, and 6% clay (loamy sand) belonging to Vertisols (Order) as Usterts (Suborder) [16]. The wheat agriculture at this location spans an area of approximately 8 hectares, with a control site surrounding it with a perimeter of 3 m wide.

### 2.2. Soil Sampling

**Figure 1:**
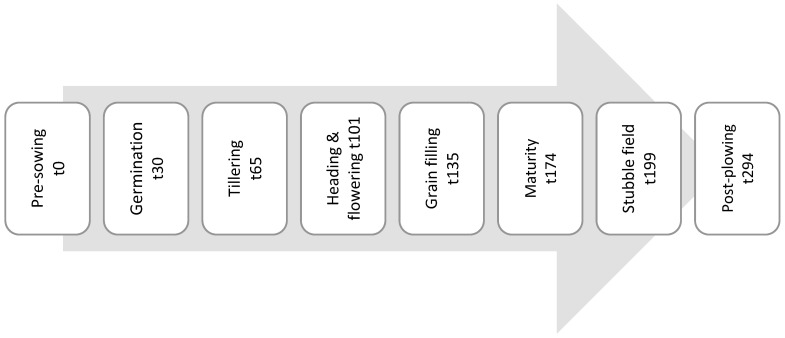
Soil sampling events according to wheat plant phenology: pre-sowing, germination, tillering, heading and flowering, grain filling, maturity, stubble field, and post-plowing.

Surface, 0 to 10 cm soil samples were collected from an area of roughly 300 by 300 m, by using a completely randomized block design sampling technique. A composite sample of 5 replicates and soil samples was collected from each field at each sampling time: the wheat field (WF) and the control site (CO). The soil samples were obtained from the wheat plant roots (from the rhizosphere) during each growing phase of the wheat plant’s growth cycle, spanning a total of seven sampling periods, (a total of 35 samples from wheat and 35 samples from control) from pre-sowing (t0) to post-plowing (t294), as depicted in Figure 1. Given the characteristics of wheat roots and their density, the soil samples were taken from the vicinity of the plant base, representing the rhizosphere. The collection of soil samples commenced in November 2020, before the wheat sowing, and continued until August 2021, after the harvest. Soil sampling was conducted during the early morning hours. Each replicate from every sampling site was carefully placed in an individual plastic bag and then transported to the laboratory inside an insulated container to prevent any temperature-related effects. During the wheat plant growth, at every sampling event, five replicates of five whole *Triticum aestivum* wheat plants were excavated randomly.

Plants were dried at 105 °C for 4–5 days, cleaned of soil particles, and then weighed to determine the total aboveground and belowground biomass [17].

Upon reaching the laboratory, each soil replicate was subjected to sieving with a 2 mm mesh size to eliminate root particles and other organic debris. The sieved soil samples were then stored at 4 °C until both abiotic and biotic analyses were carried out. All analyses were completed within 14 days of sampling. Before MicroResp™ (ABgene, Aberdeen, UK) experiments, soil samples were acclimatized for 48 h at 26 °C while being adjusted to moisture contents equivalent to 40% of their WHC (Water Holding Capacity).

### 2.3. Abiotic Soil Analysis

All chemical and biological analyses were conducted on each of the replicates collected in the field. The rainfall data were obtained from four meteorological stations using automatic rain gages located at the field site.

Soil moisture (SM) was determined gravimetrically using 10 g of fresh soil collected from the wheat rhizosphere and from control site, which was weighed and dried at 105 °C for 48 h and was expressed as a percentage of dry weight [17]. Organic matter (OM) content was determined by igniting the dry soil samples at 400 °C for 6 h [18]. Soil pH was determined using a pH electrode in a filtered supernatant (soil:double distilled water = 1:2), followed by shaking for 10 min (160 rpm), overnight incubation at room temperature, and filtration with two sheets of filter paper [3]; IV). Electric conductivity was measured by an EC meter in a filtered supernatant (soil: double distilled water = 1:10), followed by shaking for 30 min (160 rpm), overnight incubation at room temperature, and filtration with two sheets of filter paper [19].

### 2.4. Soil Biotic Parameters

CO_2_ evolution and biomass of the microbial population in the soil, as well as the functional diversity and their physiological characteristics, were determined according to the MicroResp™ plate method [15,20]. This method is based on the principle of testing the change in carbon dioxide emissions as a characteristic of the functional composition of the microbial population. It consists of a dye-plate colorimetric reaction using an absorbent alkali with the ability to absorb CO_2_ released from the samples [20,21]. Fifteen different carbon sources that characterize soil populations were selected based on four groups: amino acids, carbohydrates, carboxylic, aromatic acid, and a control solution consisting of water as described in [21]. The active soil microbial community was estimated by converting microbial glucose-induced respiration rates to biomass [22]. The result for each well was calculated and compared to the 16th well (soil amended only with water), measuring the basal respiration with no added carbon source.

Microbial functional diversity was determined using the Shannon–Weaver index (H′): H′0 − ΣPi (ln Pi), where Pi is the ratio of the activity of a particular substrate and the sum of activities of all substrates [23]. The metabolic quotient (qCO_2_), expressing the ratio between basal respiration and microbial biomass as correlating positively with energy demand and hence negatively with carbon-use efficiency of soil microbial communities, was also calculated [24]. The metabolic quotient (qCO_2_) of the microbial community correlates positively with energy demand, and hence negatively with the carbon-use efficiency of the soil microbial communities. It is widely accepted that qCO_2_ is elevated when a microbial community operates inefficiently and diverts a higher proportion of carbon to maintenance requirements than to biosynthesis as a result of an exogenous disturbance [25,26]. Division of the basal respiration rate by the respiring biomass provides the metabolic quotient (qCO_2_).

Comic/Corg—microbial coefficient ratio—was determined as the ratio between microbial biomass and total organic carbon (TOC). It is used as an indication of system equilibrium, reflecting substrate availability throughout the study [22,27].

### 2.5. Statistical Analysis

All data were subjected to statistical analysis of variance using the SAS/STAT (Software 3.3) (ANOVA Duncan’s multiple range test, and Pearson correlation coefficients) and were used to evaluate differences between separate means. ANOVA was followed by Tukey’s honestly significant difference test to establish the significance of differences between plot areas using the Statistica statistical package. Differences obtained at levels of *p* < 0.05 were considered significant.

## 3. Results

The total registered cumulative precipitation throughout the research, from November 2020 to August 2021, was 594.2 mm. Around 38% of this precipitation occurred during the pre-sowing phase (T0) (Figure 1).

A significant 94% of the total during-growth rainfall was recorded during the period from the start of growth to the beginning of seed ripening (t135), playing a crucial role in facilitating the growth and development of wheat plants. There was a notable decrease in the amount of precipitation from t135 to the end of the study at t294, covering the seed ripening phase and continuing beyond plowing.

The soil moisture content during the rainy season closely mirrored the precipitation patterns, exhibiting fluctuations between 2.8% and 20.2% (Table 1). There were no significant differences in soil moisture levels between the wheat field and the control area (*p* > 0.05). Moreover, at various points along the phenological axis (t0, t174, t199, t294), no significant variations were observed in soil water percentages among the different study areas, specifically at t101 and t135. During the germination stage (t30), the soil water content was significantly higher (*p* < 0.05) compared to all other phenological stages. A subsequent peak in water content was noted during the blooming stage (t101). A notable difference (*p* < 0.05) in soil organic matter content was observed between the wheat field and the control area throughout all eight phenological stages. This difference is attributed to the accumulation of organic matter in the wheat field. Specifically, during the pre-sowing stage (t0), the soil organic matter content was significantly higher (2.8%), *p* < 0.05), surpassing levels recorded in other phenological stages. At the sowing stage (t65), the recorded organic matter content decreased to 1.1%.

The pH levels fluctuated between 7.6 and 8.1 in both sampling sites. The wheat growing area (WF) consistently showed slightly higher pH levels compared to the control area (CO) throughout the entire study period. However, it is important to note that these differences were not significant (*p* > 0.05) throughout the study period.

Variations in pH were found between the different growth stages, where the pH at t0 was significantly (*p* < 0.05) higher compared to t135 in the control sampling site.

### 3.1. Plant Biomass

**Figure 2:**
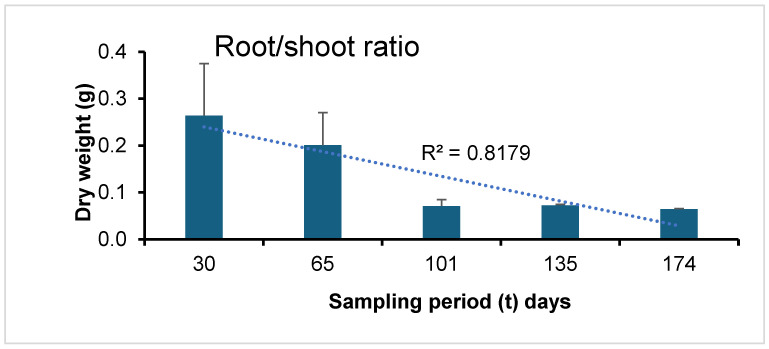
Root/shoot ratio along the phenological stages. The dotted line represents the linear correlation.

The biomass data for wheat plants demonstrated a consistent growth pattern throughout all phenological stages, ranging from 0.62 to 6.36 g per plant, encompassing both aboveground and belowground dry biomass. While the germination stage at t30 and the establishment stage at t65 showed a relatively modest increase in plant biomass, a significant increase was observed from the flowering stage at t101 onward. Moreover, a strong correlation was identified between the final aboveground and belowground biomass, with a correlation coefficient (r) of 0.89, signifying a robust relationship and affirming the reliability of the obtained results. The proportion of belowground relative to aboveground biomass increased over the phenological period (Figure 2), with a correlation coefficient (r^2^) of 0.82, underscoring the strength of this correlation.

### 3.2. Microbial Community

#### 3.2.1. Microbial Biomass

**Figure 3:**
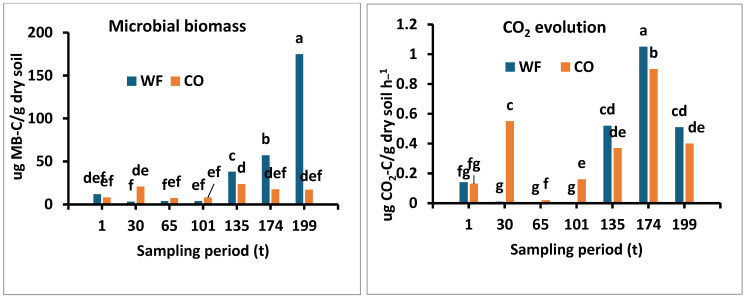

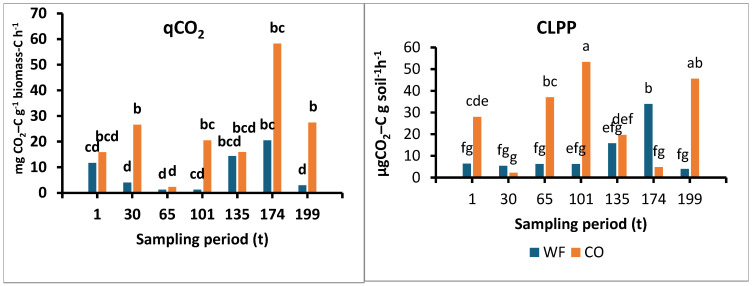
Changes in the mean soil microbial biomass, CO_2_ evolution, qCO_2_, and CLPP at two research sites: a wheat field (WF) and an uncultivated control soil (CO), throughout the wheat growing period. Small letters indicate significance (*p* < 0.05).

No significant differences in soil microbial biomass (MB μg C·g soil^−1^) C determined by glucose were found in the first 101 days in both soil samples (Figure 3). The microbial biomass C increased to 57.1 μg C·g soil^−1^ in the heading and flowering phenological stage (101 t) and then increased threefold to 174.7 μg C·g soil^−1^ (*p* < 0.05). The MB of control soil ranged between 8.0 and 23.7 μg C·g soil^−1^, where the ratio between the two sampling sites in the stubbled field (199 t) was 10-fold greater.

#### 3.2.2. Microbial CO_2_ Evolution

The figure (Figure 3) represents the CO_2_ evolution (µg CO_2_-C g^−1^ h^−1^) from soil samples collected from both sites. The CO_2_ evolution in the control samples was highest in the first 101 days and was associated with the rainfall events. At the onset of the first rain (t0), the CO_2_ evolution was similar, but after day 101, the differences between the two sites increased significantly (*p* < 0.001) in the wheat field. The rate of CO_2_ evolution from the wheat field increased significantly (*p* < 0.05) compared to the control site until the stubble field phenological stage. No significant differences were observed in CO_2_ evolution between the sites after the stubble stage, at the post-plowing stage (t199), and entering the dry season.

#### 3.2.3. Metabolic Quotient (qCO_2_)

The metabolic quotient (qCO_2_) (mgCO_2_-C(g Cmic)^−1^) reflects the CO_2_ evolution per unit of MB and indicates the maintenance requirements for biosynthesis as a result of an exogenous disturbance. As can be seen from Figure 3, the general trend along the wheat phenological stages in the control soil samples was significantly higher (*p* < 0.05) compared to the wheat field soil samples. The significant differences ranged from 1.23 to 20.5 mgCO_2_-C (g Cmic)^−1^ in the wheat field soil samples and from 20 to 58 mgCO_2_-C(g Cmic)^−1^ in the control field samples, an increase of 3–22-fold, which was associated with the effects of environmental changes on the microbial community. The increase in qCO_2_ in the control site relative to the wheat field reflects the ecophysiological status of the microbial community, with a higher CO_2_ evolution per MB unit.

#### 3.2.4. CLPP

The average values of the CLPP measurements taken at t30 and t174 were observed to be low, with some resemblance to the control sampling sites. In numerous instances, changes in the quality of C substrates availability due to environmental factors can impact the CLPP.

#### 3.2.5. C_mic_/C_org_ Ratio

C_mic_/C_org_—microbial coefficient ratio—reflects substrate availability (Table 1). Along the phenological stages, the microbial coefficient changed dramatically within the two sampling sites, where a significant difference (*p* < 0.01) was obtained at the germination (t30), tillering (t65), and heading and flowering (t101) stages. The significant decrease in Cmic/Corg at the wheat field was dramatically higher compared to the control site, reflecting the requested availability ratio of organic carbon, which disappeared.

#### 3.2.6. Correlation Between Abiotic Variables and Biotic Components

Pearson correlations between the abiotic and biotic components in the wheat sampling site (Table 2) yielded significant negative correlations between SM-EC, where the biotic components were significantly affected (*p* < 0.01) by SM–MB *p* < 0.001, SM–CO_2_ evolution (*p* < 0.0001) throughout the study period. The relation between the OM was significantly correlated with pH, EC, and MB. The biotic measurements were related to MB–CO_2_, qCO_2_–CO_2_. Substrate utilization was correlated significantly to CO_2_ evolution and qCO_2_. All the above correlations (Table 2) elucidate the interaction between the abiotic and biotic components.

At the control site, Pearson correlations indicated that the strongest significant relationship was between SM and EC, with no other correlations (Table 2).

#### 3.2.7. Substrate Utilization (SIR)

The substrate-induced respiration results in CO_2_ evolution were measured after amending the soil samples with available nutrients: fifteen substrates belonging to four groups (aromatic, carboxylic, carbohydrates, and amino acids) that are mainly present in the soil system were used in order to represent the microbial community substrate utilization expressed by CO_2_ evolution.

After that period, the microbial biomass in the soil in the wheat field remained stable until t101, contrary to the control area where a decrease was observed. Starting from t135, the month of March, the microbial biomass in both study areas began to increase, especially in the wheat field, until a peak was observed at t199 (174.7 μg C g^−1^ soil). A significant decrease in the average microbial biomass values was observed at the end of the study period (after tilling the soil in the wheat field).

In the SIR tests, several notable differences in the utilization of the carbon substrates were observed. In soil samples collected from the wheat field and from the control area, the maximum utilization of the carbon substrates was associated with the carboxylic acid group. In the wheat field, peak utilization occurred at t174 (full ripening) and in the control area at t65 (establishment). Utilization of amino acids in the control field was more significant compared to the wheat field during all phases of the study.

**Figure 4:**
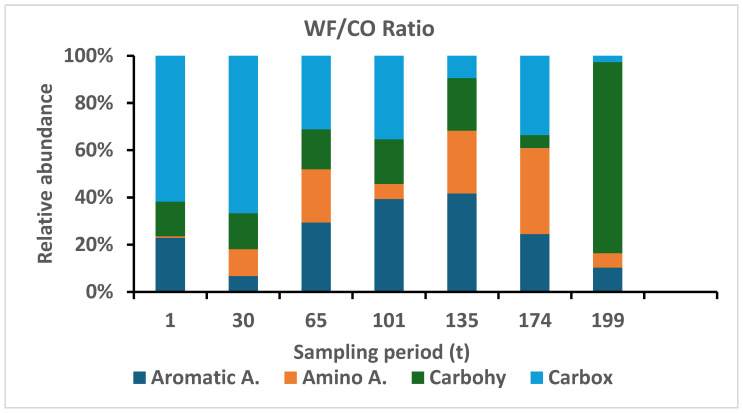
Wheat field (WF) soil substrates utilization relative to control (CO) soil samples.

Utilization of aromatic acids by microbial community in the wheat field soil in comparison to the control site was significantly different at t0, where such difference disappeared starting from t30 (germination) and no significant changes were measured until the end of the study period. Moreover, utilization of carbohydrates in the wheat field samples was significantly higher from day t135 until the end of the sampling collection, contrary to the control sampling site where a constant utilization of carbon was measured (Figure 4).

Pearson correlations between the two abiotic and each of the four substrate groups in the wheat sampling site (Table 3) indicated significant negative correlations between SM, OM, and the four substrates. The pH had a negative and significant (*p* < 0.05) correlation with each of the four substrates consumption. A significant correlation was also obtained between each substrate, emphasizing the importance of fusion between two or more substrates for more efficient nutrient assimilation.

At the control site, the OM and pH significantly (*p* < 0.05) affected the amino acid substrate uptake. pH also significantly (*p* < 0.05) affected the uptake of carbohydrates and carboxylic acid. Pearson correlations yielded the strongest significant correlation between aromatic and carboxylic acid at the control site soil samples (Table 3).

**Figure 5:**
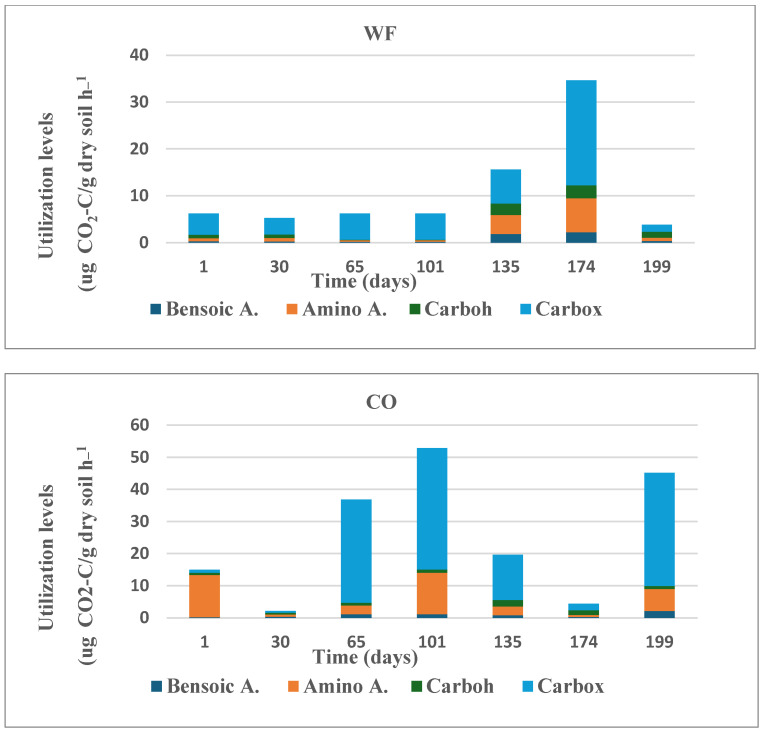
Utilization of carbon-based substrates divided into four different groups: aromatic acids, carboxylic acids, carbohydrates, and amino acids from the two sample areas: WF (wheat field) and CO (control uncultivated soil).

The proportional utilization of carbohydrates was relatively stable during the growing season (Figure 5), where at the end of the phenological stage the rate reached a maximum in the wheat field, elucidating the importance of rhizosphere preservation of nutrient (sugars and starches) that are used by microorganism in both structural and metabolic roles.

Amino acids in the plant rhizosphere can be absorbed by plant roots and bypass the microbial mineralization of organic nitrogen, which is reflected in Figure 5, where the relative utilization was low, and reached 0.027 µg CO_2_-C g^−1^ h^−1^ of the total substrate use at the heading and flowering (t101) stage. At t135 and t174, the percentage of amino acid increased to 27 µg CO_2_-C g^−1^ h^−1^, a 1000-fold increase compared to the previous stage.

**Figure 6:**
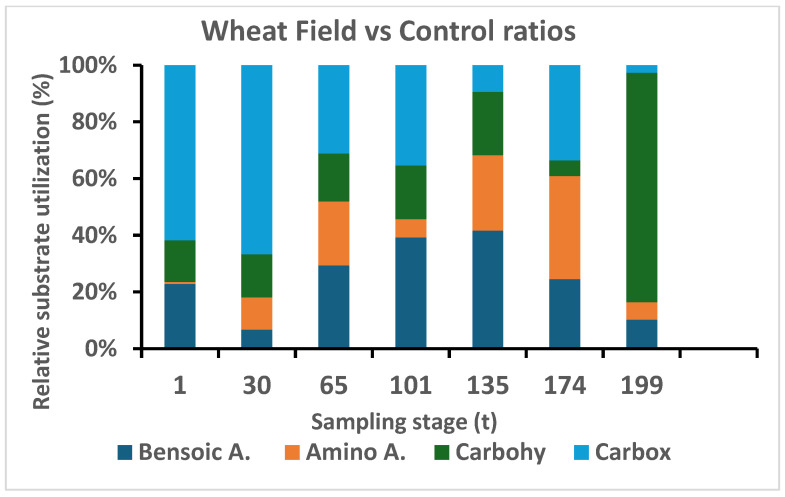
Changes in relative substrate use (WF/CO) by the microbial community of each of the four substrates along the phenological development of wheat plant (WF).

The carboxylic acid level was relatively high, 4.53 µg CO_2_-C g^−1^ h^−1^ at time zero (t1), and increased to a maximum of 22.4 µg CO_2_-C g^−1^ h^−1^ (Figure 5, WF, CO) in the control fields as well. However, the ratio (Figure 6) indicated the significance of its increase in the wheat field relative to control soils due to its inability to increase the ability to favor the two citric acid transports of complex nutrients such as Ca^+2^, Mg^+2^, Fe^+2^, etc.

The changes in benzoic followed the phenological development of wheat, increasing from 0.64 µg CO_2_-C g^−1^ h^−1^ to 2.3 µg CO_2_-C g^−1^ h^−1^ from day 30 to day 135, followed by a decrease. Benzoic acid is known to play a bioregulator role in chlorophyll a and b and stimulates plant health by being a complementary nutrient source.

#### 3.2.8. Physiological Profile (CLPP)

The community-level physiological profile (CLPP) (Figure 6) was assessed by different carbon substrate utilization abilities by the biota soil community in soil samples. By supplying different substrate sources, we were able to compare their relative exploitation consumption by microfloral components of the different groups. The CLPP of the microbial community was significantly different (*p* < 0.01) in the wheat field in general, and the phenological stage had a significant (*p* < 0.002) effect, generating a significant difference between the two sites, with values of 6.2 µgCO_2_–C g^−1^ soil h^−1^ and 6.4 µg CO_2_–C g^−1^ soil h^−1^ for the first four phenological stages, where on day t135 it increased to 15.8, followed by an increase to 33.9 µg CO_2_–C g^−1^ soil h^−1^ on day t174. An opposite trend was found for the control site, where only two cases, the 30t and t174 values, were low (2.2 µg CO_2_–C g soil^−1^ h^−1^), reaching values between 19.7 and 53.3 µg CO_2_–C g^−1^ soil h^−1^ at t101.

**Figure 7:**
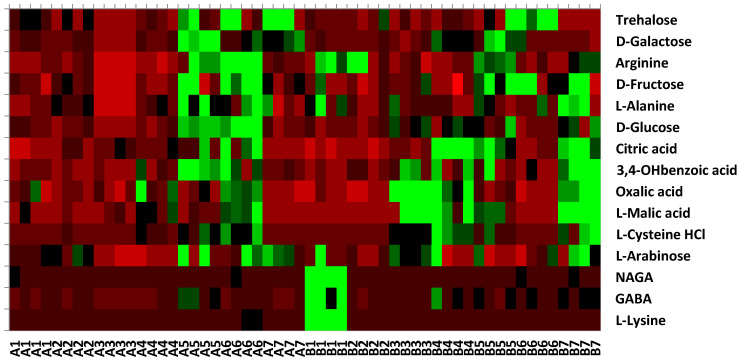
Clustered heatmap of the differential microbial activity for fifteen substrates for each of the phenological stages, A—wheat field and B—control field sampling sites (1–7 phenological stages, 1—pre-sowing; 2—germination; 3—tillering; 4—heading and flowering; 5—grain filling; 6—maturity; 7—stubble field).

The responses of microbial activity to fifteen substrates for each of the phenological stages are presented in Figure 7. The positive responses presented in the heatmap are scattered along the phenological stages for each sampling site. The highest CO_2_ evolution in both soil sampling sites is represented using carboxylic acid (Figure 7; Table 4), whereas in the wheat field, it was intensively used all along the wheat growth, while in the control (CO) field high values appeared on days t65, t101, t135 and t199 and not on days t0 and t30. Amino acids were the second group of substrates that were highly utilized in the control site (Table 4) on all the sampling days. Soil microbial community main substrate utilization in the wheat soil samples occurred at t135 and t174, the grain filling and maturity stages, respectively.

## 4. Discussion

The Mediterranean climate changes the hydrological cycle by changing both the amount and distribution of precipitation, and this is especially notable affecting wheat phenological development. Rainfall precipitation is essential in regulating soil biota community activity, as it directly influences soil moisture, nutrient availability, and plant growth. The timing and volume of rainfall have a significant impact on microbial and macrofaunal populations, which in turn affects soil structure, fertility, and the cycling of crucial nutrients. A clear understanding of these dynamics is imperative for implementing sustainable agricultural practices and effective land management strategies [28]. Moreover, these unpredictable variations in the precipitation amount and its dispersion are affecting soil moisture levels, soil biota community activity, and nutrient supplies to the above-ground primary producers. Several studies have reported that the composition of the soil microbial community in the plant rhizosphere varies along with plant phenology [29], indicating that plant roots induce distinct selective pressures, and thus select different microorganisms, at different stages of development, most likely by changes in the composition of the root exudate [30,31,32]. The present study found that the spatial heterogeneity of soil respiration across the wheat rhizosphere samples was not significant and notably different from the control soil samples, a constant along the phenological development in the wheat rhizosphere; these results were mainly related to the different abiotic components.

*T. aestivum* shows a higher root-to-shoot ratio during its early growth stages than in its elongation and reproduction phases. This shift indicates that the plant initially prioritizes root development to enhance nutrient and water absorption [33]. The largest microbial biomass, throughout the study, was measured between t135, the seed filling phase, to a peak at t199, the post-harvest, before the tillage phase, correlating to the maximum plant residues biomass. The relationship between crop residues and microbial communities’ biomass and functions is well reported [34].

During the wheat growth stages at maturity (t174) and in the stubble field (t199) in the wheat field, there was a significant increase in the utilization of four substrate groups: benzoic acid, amino acid, carbohydrates, and carboxylic acid (see Figure 5). After the stubble field stage, there was a sharp decrease in the utilization of all four substrates. In the control site, relatively high values were observed at t65, t101, t135, and t174, before harvesting. This result is similar to a study on agricultural wheat soils that were fertilized with high nitrogen, suggesting that even in the soil tested in our studies, the percentage of nitrogen in the soil was high. In the wheat field, when the utilization of carboxylic acids decreased, the utilization of carbohydrates increased, which is consistent with a previous report [35]. Additionally, as also demonstrated by [36], the utilization of amino acids was higher in the control field compared to the wheat field. Studies that examined soil biochemical indicators at different wheat growth stages under varying tillage and straw management practices found that soil organic carbon (SOC) content and microbial biomass were highest at specific growth stages, suggesting that wheat development stages significantly impact soil microbial dynamics [37]. The difference in the utilization of the various substrates in the two study areas indicates the diverse metabolic capacities of the microbial communities in the soil. It can also be inferred that when researchers tested the utilization of substrates in soils with high nitrogen content, they found that nitrogen fertilizer damaged the ability of the soil microbial communities to utilize substrates based on amino acids, which supports our results [38].

The study found that two of the 15 substrates tested, L-malic acid and D-galactose were only used once during the wheat plant’s growth stages: at maturity (t174) and grain filling (t135), respectively. Seven substrates were again irregularly used, twice, during grain filling and maturity stages (refer to Table 4). Additionally, trehalose and L-arabinose were utilized at three sampling periods: grain filling, maturity, and stubble field. Trehalose plays a crucial role in helping wheat plants survive extended periods of low soil moisture, preventing desiccation. On the other hand, arabinose serves as a carbon source for the production of organic acids and plays a role in determining plant cell wall structure. The presence of these substrates in the soil corresponded to the belowground wheat plant biomass growth rhythm.

To be able to improve our understanding of wheat rain-fed production future studies should focus on exploring the long-term impact of trehalose and L-arabinose on wheat resilience under varying soil moisture conditions. Furthermore, investigating the specific mechanisms by which arabinose affects cell wall structure and organic acid production would provide valuable insights.

Wheat production in rain-fed regions must be enhanced by adopting effective management practices. It is essential to infiltrate and conserve moisture in the soil, select the most suitable wheat cultivars, and time the sowing accurately according to recommended methods. The judicious use of fertilizers is imperative, along with diligent pest examination of the interactions between these substrates and other soil nutrients could enhance our understanding of their role in below-ground biomass growth and weed control. Moreover, proper harvesting techniques are crucial, and preventing aquifer pollution is non-negotiable to ensure the sustainability of this vital crop.

Understanding the various phenological stages of wheat is crucial for preparing for the impacts of future climate change [39,40]. This understanding may necessitate shifts in the single-cultivar concept, as well as adaptations related to vernalization and photoperiod. Additionally, soil microbial community studies will influence changes in thermosensitivity during these phenological stages. As a result, there is a need for the development of a quick agronomical tool to support effective tillering. These studies collectively underscore the dynamic interplay between wheat growth stages and soil microbial communities. They emphasize the need for integrated management practices that consider plant development stages to optimize soil health and crop productivity in rain-fed agricultural systems.

## 5. Conclusions

The high abundance of fine wheat roots in the soil during the maturity and post-harvest stubble stages likely contributes to the increased activity and richness of copiotrophic bacterial communities. These changes in belowground biomass, followed by the deposition of aboveground organic matter, represent critical biotic factors influencing the composition, metabolic activity, density, and diversity of soil microbial communities. Our findings underscore the substantial impact of limiting abiotic factors on wheat phenology and their cascading effects on microbial substrate utilization in rain-fed *Triticum aestivum* agroecosystems. Furthermore, this study highlights the spatial heterogeneity of soil respiration and its strong correlation with plant developmental stages. Future research should focus on elucidating the mechanistic pathways underlying plant–microbe interactions under varying environmental constraints, integrating multi-omics approaches to better characterize microbial functional responses. Additionally, long-term investigations are needed to assess the resilience of these microbial networks to climatic variability and their implications for sustainable agricultural management.

## Figures and Tables

**Table 1:** Changes in mean soil moisture (%), organic matter (%), pH, electric conductivity (µS cm^−1^), and microbial coefficient ratio (Cmic/Corg) along the phenological development of wheat plants in the wheat field (WF) and control (CO) study sites. The small letters following the means represent significant (*p* < 0.05) differences between different sampling times. The sampling Time (t) are in days.

Sampling Time (t)	Soil Moisture		Organic Matter		pH		Electric Cond.		Cmic/Corg	
	WF	CO	WF	CO	WF	CO	WF	CO	WF	CO
0	3.89 d	3.87 d	2.7 a	2.0 bc	8.1 a	7.9 ab	99.9 ab	98.95 ab	5.07 d	6.37 c
30	20.18 a	20.17 a	2.0 bc	1.8 bcd	7.8 bc	7.8 bc	68.1 c	78.8 ab	0.5 e	29.73 b
65	12.46 c	10.01 d	1.1 f	0.5 g	7.9 ab	7.9 ab	67.05 c	80.9 ab	0.01 e	4.35 c
101	12.46 ab	17.99 b	1.1 b	1.9 bcd	7.9 ab	7.9 ab	67.0 bc	81.2 ab	0.01 e	8.42 c
135	13.19 c	12.75 c	1.4 def	1.4 ef	7.8 bc	7.6 c	79.3 bc	84.0 ab	35.37 b	26.43 b
174	3.34 d	3.39 d	1.5 def	1.2 ef	7.8 bc	7.6 c	76.1 bc	101.2 ab	66.88 a	69.77 a
199	3.18 d	2.88 d	2.9 a	1.7 def	8.0 ab	7.9 ab	112.5 a	116.43 a	17.59 c	23.26 b

**Table 2:** Pearson correlations between abiotic factors and relative abundance of biotic components—microbial biomass (MB), CO_2_ evolution, qCO_2_, and CLPP, from the two sampling areas: WF (wheat field)—and CO (control uncultivated soil). SM—soil moisture; OM—organic matter; EC—electric conductivity. Significance level alpha * *p* = 0.05, ** *p* < 0.01, *** *p* < 0.001, **** *p* < 0.0001.

Wheat Site (WF)							
Variables	SM (%)	OM (%)	pH	EC (µS cm^−1^)	MB	CO_2_	qCO_2_
SM (%)							
OM (%)	−0.27						
pH	−0.31	0.44 *					
EC (µS cm^−1^)	−0.39 *	0.64 ***	0.26				
MB	−0.58 **	0.44 *	0.1	0.53 **			
CO_2_	−0.60 ***	−0.07	−0.37	0.13	0.51 **		
qCO_2_	−0.27	−0.1	−0.33	−0.1	−0.15	0.64 ***	
CLPP	−0.17	−0.31	−0.48 **	−0.12	−0.01	0.71 ****	0.62 ***
Control site (CO)							
Variables	SM (%)	OM (%)	pH	EC (µS cm^−1^)	MB	CO_2_	qCO_2_
SM (%)							
OM (%)	0.1						
pH	0.12	0.02					
EC (µS cm^−1^)	−0.69 ****	0.28	−0.01				
MB	0.09	0	−0.25	−0.05			
CO_2_	−0.16	0.14	−0.43 *	0.24	0.57 **		
qCO_2_	−0.26	0.32	−0.45 *	0.39 *	0.08	0.8 2 ****	
CLPP	−0.06	0.04	0.50 **	0.14	−0.44 *	−0.57 **	−0.32

**Table 3:** Pearson correlations between abiotic factors and relative abundance of substrate utilization for each of the four groups: aromatic acid, amino acids, carbohydrates, and carboxylic acid from the two sampling areas: WF (wheat field) and CO (control uncultivated soil). SM—soil moisture, OM—organic matter, EC—electric conductivity. Values from 0 with a significance * level alpha = 0.05, ** *p* < 0.01, *** *p* < 0.001, **** *p* < 0.0001.

Wheat Site (WF)							
Variables, WF	SM (%)	OM (%)	pH	EC (µS cm^−1^)	Aromatic	Amino Acids	Carbohydrates
SM (%)							
OM (%)	−0.27						
pH	−0.31	0.44 *					
EC (µS cm^−1^)	−0.39 *	0.64 ***	0.26				
Aromatic	−0.16	−0.26	−0.49 **	−0.19			
Amino acids	−0.25	−0.31	−0.49 **	−0.09	0.63 ***		
Carbohydrates	−0.39 *	−0.17	−0.44 *	0.07	0.43 *	0.80 ****	
Carboxylic acids	−0.09	−0.28	−0.40 *	−0.13	0.54 **	0.77 ****	0.48 **
Control site (CO)							
SM (%)							
OM (%)	0.1						
pH	0.12	0.02					
EC (µS cm^−1^)	−0.69 ****	0.28	−0.01				
Aromatic	−0.11	−0.14	0.36	0.23			
Amino acids	−0.21	0.42 *	0.39 *	0.18	0.05		
Carbohydrates	−0.13	−0.13	−0.39 *	0.11	−0.04	−0.06	
Carboxylic acids	0.07	−0.2	0.39 *	0.05	0.67 ****	0.09	0.07

**Table 4:** Use each of the fifteen substrates at each study site (WF—wheat field YY; CO—control xx) along the phenological stages.

	Pre-Sowing (t0)—1		Germination (t30)—2		Tillering (t65)—3		Heading and Flowering (t101)—4		Grain Filling (t135)—5		Maturity (t174)—6		Stubble Field (t199)—7	
	WF	CO	WF	CO	WF	CO	WF	CO	WF	CO	WF	CO	WF	CO
3,4-OHbenzoic acid						xx		xx	YY	xx	YY			xx
Citric acid								xx	YY	xx	YY			xx
L-Malic acid						xx		xx			YY			xx
Oxalic acid						xx	YY	xx			YY			xx
D-Fructose									YY	xx	YY	xx		xx
D-Galactose									YY	xx				
D-Glucose									YY	xx	YY	xx		
L-Arabinose		xx						xx	YY		YY		YY	xx
Trehalose									YY	xx	YY	xx	YY	
GABA		xx												
L-Alanine		xx							YY		YY			xx
L-Cysteine HCl								xx	YY		YY			xx
L-Lysine		xx												
NAGA		xx												
Arginine		xx		xx					YY	xx	YY			
Total number of substrate used	0	6	0	1	0	3	1	6	10	7	11	3	2	8

## Data Availability

The original contributions presented in this study are included in the article. Further inquiries can be directed to the corresponding author.
